# Short Oxygen Plasma Treatment Leading to Long-Term Hydrophilicity of Conductive PCL-PPy Nanofiber Scaffolds

**DOI:** 10.3390/polym9110614

**Published:** 2017-11-14

**Authors:** Sajjad Shafei, Javad Foroughi, Zhiqiang Chen, Cynthia S. Wong, Minoo Naebe

**Affiliations:** 1 Institute for Frontier Materials, Deakin University, Geelong, VIC 3216, Australia; c.zhiqiang@deakin.edu.au (Z.C.); cynthia.wong@deakin.edu.au (C.S.W.); 2 Intelligent Polymer Research Institute, University of Wollongong, Wollongong, NSW 2522, Australia; foroughi@uow.edu.au

**Keywords:** durable plasma treatment, conductive scaffold, hydrophilic PPy-coated PCL fibers, nanofiber scaffolds, tissue engineering

## Abstract

Electrically conductive scaffolds are of significant interest in tissue regeneration. However, the chemistry of the existing scaffolds usually lacks the bioactive features for effective interaction with cells. In this study, poly(ε-caprolactone) was electrospun into aligned nanofibers with 0.58 µm average diameter. Electrospinning was followed by polypyrrole coating on the surface of the fibers, which resulted in 48 kΩ/sq surface resistivity. An oxygen plasma treatment was conducted to change the hydrophobic surface of the fiber mats into a hydrophilic substrate. The water contact angle was reduced from 136° to 0°, and this change remained on the surface of the material even after one year. An indirect cytotoxicity test was conducted, which showed cytocompatibility of the fibrous scaffolds. To measure the cell growth on samples, fibroblast cells were cultured on fibers for 7 days. The cell distribution and density were observed and calculated based on confocal images taken of the cell culture experiment. The number of cells on the plasma-treated sample was more than double than that of sample without plasma treatment. The long-lasting hydrophilicity of the plasma treated fibers with conductive coating is the significant contribution of this work for regeneration of electrically excitable tissues.

## 1. Introduction

Research on scaffolds made for tissue regeneration is progressing extensively to meet the further requirements of tissue engineering. These requirements range from morphological and mechanical to biological properties. A net-shaped structure with interconnected pores is an important prerequisite for cells to grow into their desired physical shape, and for the vascularization of the ingrown tissue to take place [1]. Mechanical properties of the scaffold are required to be fully adapted to the specific regenerating tissue. The scaffold must not collapse during surgical implantation. Also, after the implantation, a patient’s regular activities must not lead to deformation of the scaffold [2]. However, biological properties of the scaffold might be the most important elements to consider, because living cells are leading the tissue regeneration.

The chemistry of the scaffolds must be suitable for interactions with cells. Bioactive cues for cells in their microenvironment are required to facilitate proliferation and differentiation. The translation of these requirements in terms of material characteristics first and foremost mandates hydrophilicity of the surface of the scaffold [3]. Generally, the materials are expected to show water contact angles lower than 65° [4], and this requirement becomes more important when synthetic polymers without binding sites and biomolecules are being used [5]. While some types of cells have shown maximum attachment on surfaces with contact angles 40°–50° [6], some others have attached better when contact angles reduced to 0° [7]. As contact angle is a result of the surface chemistry and cannot be studied independently, it is arguable as to whether lower contact angles (values close to 0°) enhances cell attachment and growth. However, the predictability of the material behavior is imperative for implants used in biomedical applications [3]. Along with a suitable biochemistry, it is also important for the scaffold to mimic the surface topography of the extracellular matrix (ECM). ECM is a supporting structure for cell adhesion and cell-to-cell communication that is made up of different proteins, such as collagen and fibronectin. These proteins form fibrous assemblies with nanoscale topographies, such as collagen fibrils with diameter range of 50–200 nm, and fibronectin components that are 2 nm–3 nm thick and 60 nm–70 nm long [5]. This is why nanostructures with at least one physical dimension in the nanometer range are of high interest for the design of the scaffolds [8]. Nanoscale features such as grooves and indentations may provide protein binding sites that can enhance cell attachment [9]. For example, roughness of the pore walls or grooves on the fibers of fibrous scaffolds have shown enhanced cell attachment and proliferation [10,11].

For tissue engineering of electrically excitable cells, conductivity of the scaffold is a property worth considering. Bone, muscle and nerve tissues have shown promising regeneration and stimulation when cultured on conductive scaffolds [12,13,14]. Inspired by the conductive substrate and the electrical signals in neural tissue, great effort has been devoted to fabrication of conductive scaffolds. Electrical conductivity of the scaffolds can be achieved by different methods; for example, by using carbonaceous materials [15,16,17,18], metals [19,20] or conductive polymers [21,22]. One of the advantages of using conductive polymers is that they are easy to process, which is because of their polymeric structure [23,24,25]. Among the conductive polymers, polypyrrole (PPy) is widely used in biomedical applications due to its ease of synthesis, high conductivity and cytocompatibility [26]. It has been used in tissue engineering and implants [27], drug delivery [28] and biosensors [29]. For example, PPy is investigated in several implants for neural prosthetics resulting in biocompatibilities as high as Teflon in cerebral cortex of rat [30]. Although ease of processing is one criterion, another important property for the scaffolds used in tissue engineering is biodegradability. PPy is not intrinsically biodegradable; however, conductive polymers can be made biodegradable using different methods, such as fabrication of biodegradable composites, modifying the polymer backbone or reducing the size of polymer chains [21].

Electrically conductive scaffolds tend to be hydrophobic. As mentioned above, cells prefer a hydrophilic environment for attachment which can be achieved by several methods. The addition of natural-based hydrophilic polymers such as gelatin, collagen and elastin can be one option. For example, poly(ε-caprolactone) (PCL), Polyaniline (PANI) and gelatin were mixed to form a hydrophilic scaffold for nerve tissue engineering [31]. Chitosan was also used in another study to form a polysaccharide hydrogel [32]. However, the addition of hydrophilic polymers led to the conductivity of these scaffolds being significantly lower than scaffolds with a purely conductive polymer coating [31,32,33,34]. For practicality of use, in this work, we developed a plasma-treated conductive scaffold with a long-lasting hydrophilicity, and evaluated it after one year. The gas used for plasma treatment, as well as factors such as applied plasma time, can affect the water contact angle, mechanical properties and durability of hydrophilicity. While use of oxidizing gases (O_2_, air, H_2_O, N_2_O) leads to organics removal and oxygen species exposure on the polymer surface, reducing gases (H_2_ and mixtures of H_2_) leads to the removal of oxidation-sensitive substances; additionally, noble gases (Ar, He) produce free radicals for further reactions [35,36].

Some plasma treatments on scaffolds have led to water contact angles close or even equal to zero [36]. However, to the best of our knowledge, the hydrophilic nature of these plasma-treated materials usually lasts for only a short period of time, which limits the application of these scaffolds. For instance, the water contact angles 10° and 37° on two plasma-treated poly(l-lactic acid) (PLLA) and poly(lactic-co-glycolic acid) (PLGA) films increased, respectively, by approximately 16° in each case just in four hours [37]. The aging is influenced by plasma treatment parameters, along with the resultant surface elemental composition [38], as well as the way the samples are stored [39]. For example, oxygen to carbon ratios under 0.2 on the plasma treated materials [38], low storage temperatures such as 20 °C or even −10 °C, and low relative humidity values have been reported to extend the hydrophilicity achieved by some plasma treatments [39]. In this paper, we demonstrate a durable hydrophilicity on a conductive scaffold that provides an enhanced surface for cell attachment. The conductivity is achieved by a coating that is expected to be superior to mixture of materials.

## 2. Materials and Methods

### 2.1. Materials

The chemicals, disposable items and cell culture supplements used in this work were purchased from Sigma-Aldrich (Castle Hill, NSW, Australia), Interpath Services (Heidelberg West, VIC, Australia) and Thermo Fisher Scientific (Scoresby, VIC, Australia), respectively, unless otherwise stated. Poly(ε-caprolactone) (PCL Mn = 80 kDa), pyrrole, dimethylformamide (DMF), tetrahydrofuran (THF), sodium p-toluenesulfonate (Na.*p*TS) and Iron(III) chloride (FeCl_3_) were purchased from Sigma-Aldrich. Human foreskin fibroblast (HFF-1 SCRC-1041) was purchased from ATCC (Manassas, VA, USA).

### 2.2. Fabrication Procedure

**Figure 1:**
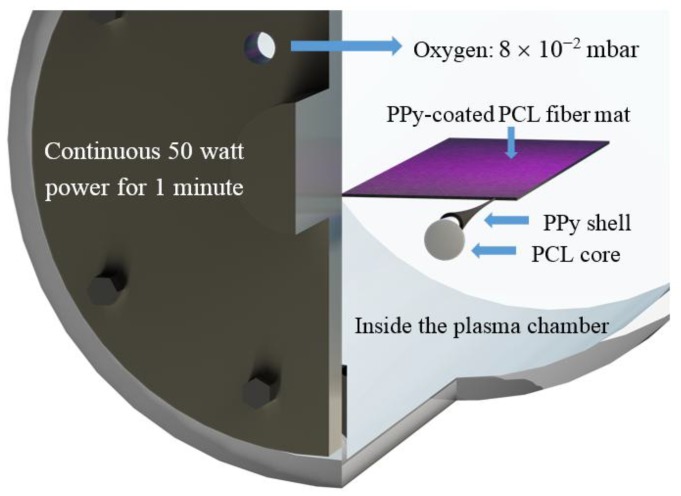
Oxygen plasma treatment of PPy-coated PCL fiber mat conducted in a plasma chamber; PCL: poly(ε-caprolactone); PPy:polypyrrole.

The PPy-coated PCL nanofibers were fabricated by firstly electrospinning PCL fibers on a drum, which were then coated with PPy and finally plasma treated using oxygen. To electrospin fibers, 14 wt % PCL was dissolved in DMF:THF with a volume ratio of 1:1 using a magnetic stirrer at 50 °C overnight. PCL fibers were electrospun with an applied voltage of 13 kV, while 2 mL/h was used as the solution flow rate. A 12 cm diameter drum rotating at a speed of 1.4 krpm was placed at 20 cm distance from the needle (18 G) to collect aligned fibers [34]. To coat PCL fibers, samples of 5 × 3 cm^2^ were dipped into a 20 mL solution of 14 mM sodium *p*-toluenesulfonate (Na.*p*TS), and 14 mM pyrrole dissolved in water. Due to the hydrophobicity of the PCL fibers, a 1 min ultrasound bath was used to make sure the entire surface of the fibers was soaked, and then the samples were left in the solution for 2 h at room temperature. The second step of coating was to add 20 mL of 38 mM FeCl_3_ solution in water to the glass container of the samples and leave the samples in the container overnight. The final step was to wash the samples thoroughly with methanol and water to remove the residuals produced during the coating process. A cylindrical plasma reactor (a purpose-built, inductively coupled radio frequency 13.56 MHz reactor) was used for plasma treatment [40]. Samples were placed in a plasma chamber under the plasma source, and were treated by continuous wave oxygen plasma with oxygen at 8 × 10^−2^ mbar. Continuous 50-watt power was used in the chamber for 1 min to conduct the plasma treatment as schematically shown in Figure 1. The optimized process parameters for a durable hydrophilic surface were found based on previous studies [41,42,43] and some trial and error. Samples were then removed, and their water contact angle was tested immediately, one month after, and one year after the plasma treatment.

### 2.3. Morphology

Scanning electron microscopy (SEM) images were taken from the surface of the fibers using Zeiss Supra 55VP (Oberkochen, Germany). The fibers were gold coated (5 nm thickness) and placed in a vacuum chamber overnight. The day after gold coating, samples were moved to the SEM imaging chamber. EHT = 5 kV were applied as the electron voltage at an approximate working distance of 5 mm–7 mm and with an aperture size of 10 µm. The fiber diameter of electrospun fibers was measured using an image per sample. Calibration of pixel size on each image was carried out in ImageJ software (1.50i, National Institutes of Health, MD, USA, 2017). Approximately 400 µm^2^ was studied for each sample, and 100 measurements were conducted in ImageJ software, with each site being labelled to prevent repeated measurement.

### 2.4. Water Contact Angle

Water contact angle of the samples was measured based on ASTM D5946-09. Droplets of 5 µL in volume were placed on the surface of the fiber mats and Young/Laplace method was used to process the images taken of the droplets. The contact angles of the droplets were observed immediately, one month after, and one year after the plasma treatment. Averages and standard deviations of these measurements were calculated and studied.

### 2.5. Chemical Analysis

X-ray photon spectroscopy (XPS) was used for the analysis of the surface composition on the scaffolds. A Kratos AXIS Nova spectrometer (Kratos Analytical Ltd., Manchester, UK), equipped with a monochromated Al Kα X-ray source (hν = 1486.6 eV), was used, and operated at 150 W. Survey spectra were acquired at a pass energy of 160 eV and at 1 eV/step. Spectra of the characteristic photoemission for selected elements (e.g., C 1 s) were acquired at a pass energy of 20 eV and at 0.1 eV/step. During the acquisitions, the samples were flooded with low-energy electrons to compensate for the accumulation of surface charge, which occurs when analyzing insulators. The pressure inside the analysis chamber was ~5 × 10^−9^ Torr. For the analysis, spectra were first calibrated by carbon peak, and then the chemical elements were detected by CasaXPS software (2.3.18, Casa Software Ltd., Kyoto, Japan, 2017). The elemental composition was determined based on the area of the peaks for each element. Finally, the high-resolution spectra for carbon and nitrogen were analyzed to fit curves for different bonds and determine the proportion of each bond based on the area.

### 2.6. Electrical Resistivity

Electrical resistivity was measured according to AATCC 76-2011 standard. Electrospun fibers were cut into 3 × 1 cm^2^ samples. Conditioned samples at 65% relative humidity and 24 °C were tested both in the spinning direction and the perpendicular direction. Two clean copper probes (width = 1 cm) were placed on the sides of 5 specimens in each direction. Pressure was applied to the copper strips to ensure contact. As specified by the AATCC standard, the pressure being applied on the probes was increased until 1.2 N/cm^2^, after which further pressure did not affect the results. The probes with 10 mm distance, were connected to a multimeter (Fluke 189) for resistivity measurements and 3 significant figures were read for each measurement.

### 2.7. Cell Culture

An indirect method was first used based on ISO 10993-5 to evaluate the cytotoxicity of the samples using human foreskin fibroblasts [44]. Fiber mats were cut to 5 × 5 mm^2^ using a surgical blade as samples for the cytotoxicity test. Samples were sterilized by first washing and then soaking them for half an hour using filtered 70% ethanol. After drying, samples were incubated in triplicate in a 48-well plate with cell culture media containing Dulbecco’s Modified Eagle Medium‎ (DMEM) + 10% Fetal Bovine Serum (FBS) + 1% Penicillin–Streptomycin for three days. Samples attached to the bottom of the wells were then removed from the media, and fourteen thousand cells were seeded in the incubated media for three days without scaffolds. Media were then removed, and the wells were washed using phosphate buffer saline (PBS). Adhered cells were then harvested from each well using 0.5% Trypsin/EDTA. Finally, dead cells were stained with trypan blue, and cell viability was calculated manually as the number of live cells to the total number of cells. Each well was compared with control wells that had no sample incubated in them.

In addition, fibroblast cells were used to evaluate plasma treated scaffolds due to existence of the bilayered structure of fibroblast and nerve cells in the regeneration site. This concept has been used in the area of nerve tissue engineering based on the formation of an outer layer of fibroblast cells and an inner layer of nerve cells [45]. Briefly, 8 × 10 mm^2^ samples were prepared in triplicate (thickness: ~300 µm) and sterilized in filtered 70% ethanol for half an hour in 48-well tissue culture plates. After washing in PBS, samples were pre-incubated in the culture media for half an hour to allow better attachment of the cells to the scaffolds. After removing the media and drying the scaffolds, fourteen thousand fibroblast cells suspended in 50 µL were seeded onto the scaffolds in wells. Then, 500 µL of media was added to each well for all samples, and they were then cultured in a humidified environment at 37 °C and 5% CO_2_. Seven days after seeding, cells were stained and observed under fluorescent microscopy. For staining, cells were fixed using 4% paraformaldehyde for 10 min and permeabilized using 0.5% Triton X-100 for 10 min. Cells were then stained with 100 nM Rhodamine Phalloidin (Thermo Fisher R415, Waltham, MA, USA) for 30 min and with 100 nM DAPI (Thermo Fisher D1306) for 1 min. The morphology of the cells was observed under Leica SP5 confocal microscopy and, where required, stack imaging was used to capture sharp and clear images of the cells. Tile scanning was used at 10X magnification to cover a wide area for quantitative analysis of the cells adhered to the scaffolds to count the cells by ImageJ software.

### 2.8. Statistical Analysis

Statistical analysis was carried out using SPSS 22.0 for Windows. Values are represented as mean ± standard deviation. Mean comparison was performed using one-way analysis of variance (ANOVA) with significance level of *p* ≤ 0.05 in the analysis

## 3. Results and Discussion

### 3.1. Morphology

**Figure 2:**
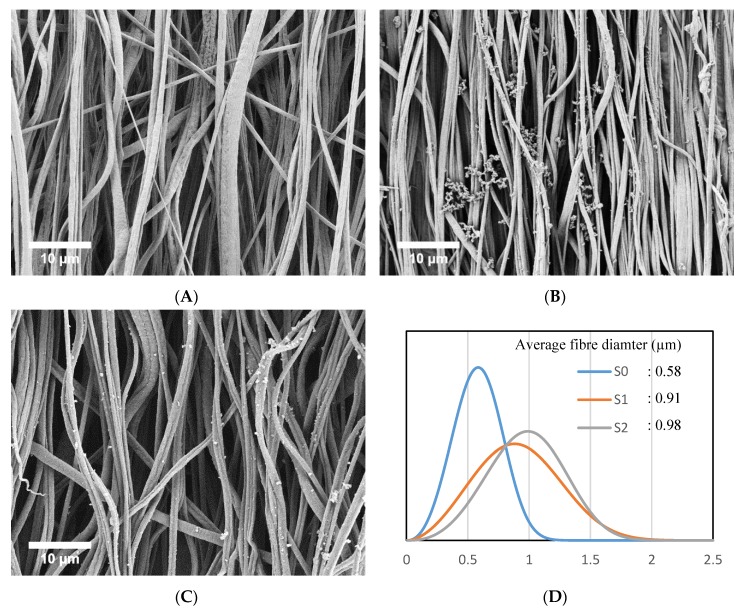
The morphology of the fibers and the fiber diameter distribution. (**A**) Aligned PCL fibers without coating (S0); (**B**) Aligned PPy-coated PCL fibers (S1); (**C**) Plasma-treated aligned PPy-coated PCL fibers (S2); (**D**) Fiber diameter distribution by Weibull curve fitting.

The morphology of PCL fibers without coating (S0), PPy-coated PCL fibers (S1) and plasma treated PPy-coated PCL fibers (S2) is shown in Figure 2A–C, respectively. Figure 2D shows the average and distribution of the fiber diameter for the samples. The coated fibers are significantly coarser than the PCL fibers without coating (*p* < 0.05, ANOVA). The fiber diameter for the plasma-treated fibers was not statistically different from non-coated fibers. This was expected, because polypyrrole as a coating has high chemical and thermal stability, and is not expected to deform during the plasma treatment [21,46].

### 3.2. Water Contact Angle

**Figure 3:**
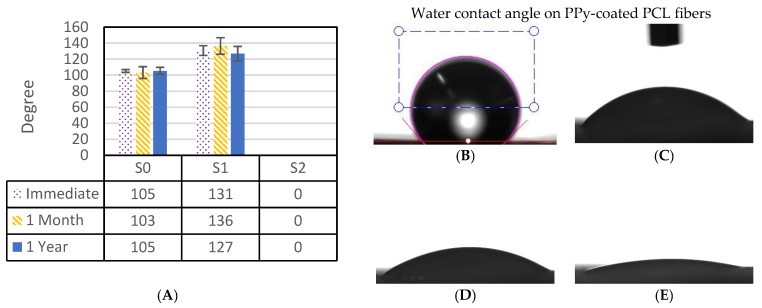
A. Water contact angle on pure PCL fibers (S0) and on coated samples. Image B shows the water contact angle of PPy-coated PCL without plasma treatment (S1) after it stabilized (~1 s), while images (**C**–**E**) shows the change of a water drop for the plasma-treated sample (S2) over time in a timeframe of 0.01, 0.03 and 0.05 s. The water contact angle for plasma-treated samples reached 0° in less than 0.1 s. (**A**) Water contact angle in degrees; (**B**) No plasma treatment, S1; (**C**) Plasma treated, S2: 0.01 s; (**D**) Plasma treated, S2: 0.03 s; (**E**) Plasma treated, S2: 0.05 s.

The water contact angle for the samples immediately, one month after, and one year after fabrication is shown in Figure 3. The contact angles for both PCL and PPy-coated PCL fibers are higher than 100°, indicating that these samples were hydrophobic, while 0° on plasma-treated sample within 0.1 s indicates a hydrophilic surface for the plasma-treated fibers. The oxygen plasma treatment has introduced polar functional groups to the surface of the treated fibers, leading to hydrophilicity of the plasma treated sample. Samples were stored in air-tight containers at room temperature, and the water contact angle of zero degrees did not change even one year after the plasma treatment, suggesting that the hydrophilicity can be maintained for this duration. This may be attributed to permanent surface structure changes as a result of oxygen etching, or the formation of durable polar functional groups as the possible consequences of no argon cleaning for surface activation, or the short-term and low-power oxygen plasma treatment used in this study [41,47]. Previous studies with various polymers and plasma treatment conditions have shown contact angles increasing over time [37,48,49,50].

### 3.3. Chemical Analysis

**Figure 4:**
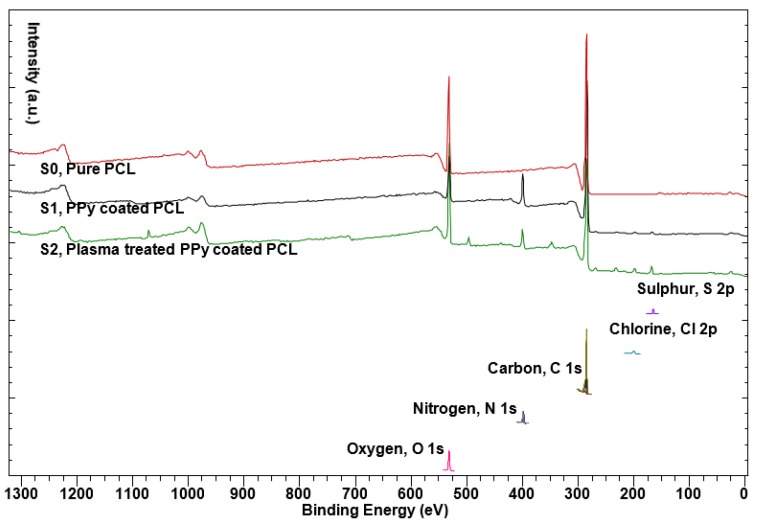
XPS wide spectra for PCL, PPy-coated PCL without and with plasma treatment. Nitrogen, chlorine and sulphur peaks are available for the coated samples. There is some contamination of plasma-treated sample, which is expected to be due to the contamination of the plasma chamber.

Figure 4 shows the XPS wide spectrum of pure PCL (S0), PPy-coated PCL without (S1) and with plasma treatment (S2). Table 1 shows the elemental composition of the samples calculated based on the XPS spectrum. As expected, nitrogen, which was used to distinguish between the coated and uncoated samples, was detected on PPy-coated fibers [51]. Also, since the polymerization process of PPy involved the incorporation of Cl and pTS, these elements were also detected in the spectra. Iron atoms were not detected in the spectra for coated samples, which is in agreement with other studies, where it is stated that the amount of Fe is below the XPS detection limit in washed samples [52]. There was some Na and Ca detected in the spectra produced by the plasma-treated fibers. This is due to the contamination of the chamber during plasma treatment, which had been excluded from the elemental composition in Table 1.

**Figure 5:**
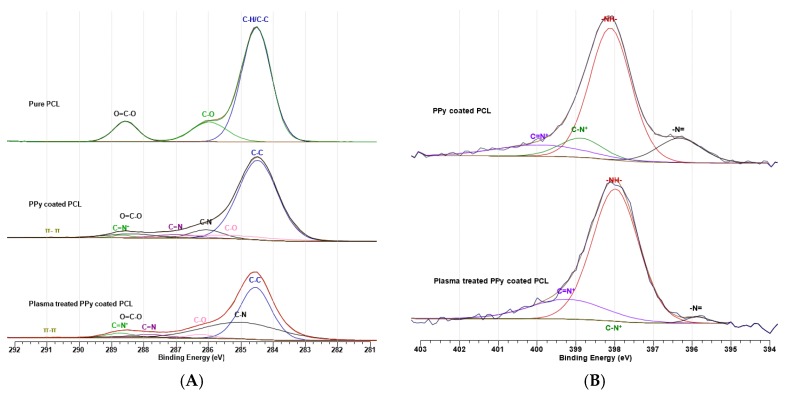
High-resolution XPS spectra of the coated and uncoated samples at C 1 s, and comparison of the coated samples without and with plasma treatment at N 1 s. (**A**) High-resolution C 1 s spectrum for PCL and PPy-coated PCL fibers without and with plasma treatment; (**B**) High-resolution N 1 s spectrum for PPy-coated PCL fibers without and with plasma treatment.

Figure 5 graphs A and B show the high-resolution XPS spectra of the samples at C 1 s and N 1 s, respectively. The carbon bond peaks of C–H/C–C, C–O and O=C–O that are in PCL both in coated and uncoated form were able to be found at 284.5, 285.3–287.5 and 288.0–289.0 eV, respectively [33,51]. Also the C–N, C=N, C=N^+^ and π–π bonds were fitted at 285–286.8, 286.2–288.6, 287.9–290.0, 289.5–291.5 eV, respectively [33,51].

In Figure 5A, considering the hydrophilicity of the plasma-treated sample, it is expected that the non-polar C–C bonds are replaced by polar bonds such as C–N. Non-polar C–C bond was reduced from 75.4% to 48.6%, while the polar C–N bond on the plasma treated sample increased from 6.7% to 38.3% (Table 2) [53,54]. Formation of these polar bonds on the surface of the fibers leads to a higher interfacial adhesion tendency for highly polar water molecules [36,37,55]. As shown in Figure 5B, the spectrum on N 1 s for PCL without coating changed to peaks with curves fitted for different nitrogen bonds on PPy-coated samples. The proportion of C–N^+^ and –N= bonds to the rest of nitrogen bonds was reduced in the plasma-treated sample, suggesting that these bonds were replaced by the more stable C=N^+^ and N–H bonds (Table 3).

### 3.4. Electrical Resistivity

**Figure 6:**
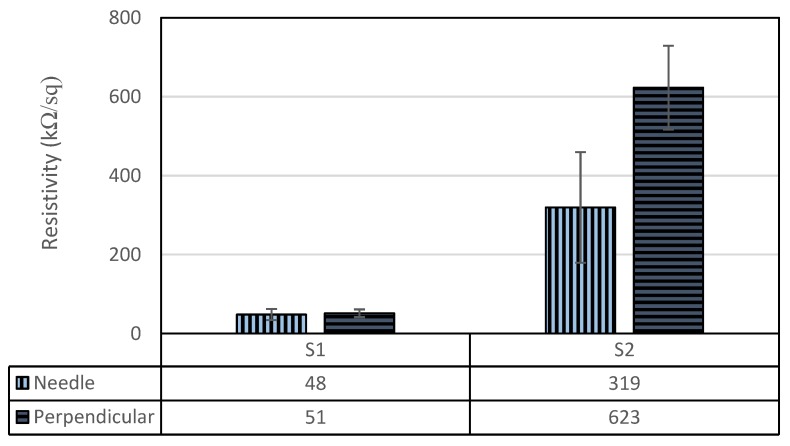
The electrical resistivity of the PPy-coated PCL fibers without (S1) and with plasma treatment (S2). The plasma treatment formed the more stable bond C=N^+^, which replaced the bonds with high electron freedom, C–N^+^ and –N=.

Figure 6 shows the surface resistivity of the coated samples S1 and S2 in both the direction of needle and perpendicular to it. After plasma treatment, the resistivity changed from 48 to 319 kΩ/sq in the needle direction, and from 51 to 623 kΩ/sq in the perpendicular direction (statistically significant under *p* = 0.0003%). As evidenced by the XPS analysis, the plasma-treated sample had considerably less capability to transmit electrons. Plasma treatment on PPy coating led to reduction of bonds with high electron freedom, i.e., C–N^+^ and –N=; therefore, when compared to S1, sample S2 was notably less conductive.

### 3.5. Cell Culture

Electrospun fiber mats were first tested in terms of cytotoxicity. Cell viabilities of 99.4 ± 0.5% and 98.8 ± 1.1% were measured for S1 and S2, respectively. No significant difference in cell viability was observed between the control wells without sample (99.7 ± 0.5% cell viability) and either S1 or S2. These results show the excellent cytocompatibility of both PPy-coated PCL and plasma-treated PPy-coated PCL fibers. This was expected, as both PCL and PPy were shown to be biocompatible in previous studies [21,56].

**Figure 7:**
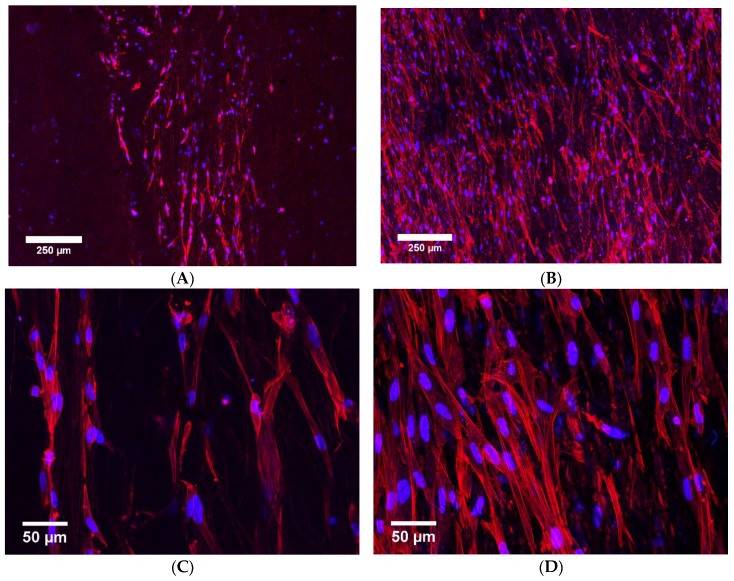
Fibroblast cells after 7 days on conductive scaffolds of PPy-coated PCL. Plasma treatment (S2) provided a better surface for the cells to attach to, compared to samples without plasma treatment (S1). This is evidenced by the cells in images B and D, which have approximately twice the cells in images A and C. (**A**) Cells on S1 at low magnification; (**B**) Cells on S2 at low magnification; (**C**) Cells on S1 at high magnification; (**D**) Cells on S2 at high magnification.

**Figure 8:**
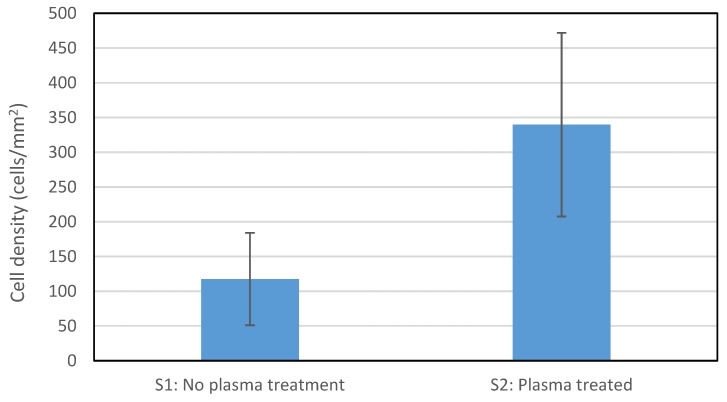
Fibroblast cell density on conductive scaffolds after 7 days.

Secondly, to compare the cell growth on PPy-coated PCL fibers, fibroblast cells grown on the scaffolds were observed after 7 days using fluorescent microscopy (Figure 7). The number of cells on the plasma-treated sample S2 (12.1 ± 3.8 thousand cells) was more than twice that on the sample without plasma treatment S1 (5.1 ± 2.3 thousand cells). Figure 8 shows the number of cells per unit area of the fiber mat. The improved cell adhesion could be due to plasma treatment changes in surface topography or chemistry with the formation of nano or micro patterns or polar bonds [57,58]. The results showed superior attachment of the cells to the plasma-treated sample (*p* = 0.024). This is consistent with previous reports about the effects of plasma treatment [36,37]. For example, plasma treatment of PCL fibers using different gases of N_2_ + H_2_, NH_3_ + O_2_ or Ar + O_2_ increased the number of attached cells after seven days by at least 30% [36]. The 0° water contact angle resulting from the plasma treatment conducted in this study contributed to better cell attachment to the hydrophilic PPy-coated PCL fibers. This has also been previously reported, whereby enhanced cell spreading and proliferation was observed as a result of oxygen plasma treatment [7].

## 4. Conclusions

Among the requirements of the scaffolds used for regeneration of electrically excitable tissues, the appropriate biochemistry of the materials is essential. In spite of many conductive scaffolds made for nerve tissue engineering, only a few of the designs contain biologically active molecules for cells to attach to. Herein, we have electrospun PCL fibers and coated them with PPy. The coated nanofibers were hydrophobic, and to provide a substrate suitable for the cells to attach to, an oxygen plasma treatment was conducted. In contrast to the majority of the plasma treatments, the water contact angle in this study did not change, even one year after the treatment, suggesting that the hydrophilicity of the fibers is permanent. From a product-development perspective, this durability in material hydrophilicity could significantly enhance the practicality of using plasma-treated scaffolds for tissue engineering. Excellent cytocompatibility was observed from the fiber mats, both with and without plasma treatment. The plasma-treated fiber mats showed a significantly better cell attachment compared to the untreated sample. The better attachment of the cells to the plasma-treated conductive fibers provides promise for tissue regeneration of electrically excitable cells.

## Figures and Tables

**Table 1:** Elemental composition of the samples by percentages.

Sample	C	O	N	S	Cl
S0, PCL	81.7	18.3	0.0	0.0	0.0
S1, PPy-coated PCL	79.1	10.2	9.8	0.6	0.3
S2, Plasma-treated PPy-coated PCL	68.6	22.6	6.3	1.7	0.9

**Table 2:** The percentage of different bonds at C 1 s spectra for pure PCL, PPy-coated PCL and plasma-treated PPy-coated PCL. The amount of non-polar C–C bonds on PPy-coated PCL was decreased after plasma treatment from 75.4% to 48.6%, while the polar bonds of C–N and C=N^+^ were significantly increased.

Sample	C–C	C–O	C=O–C	C–N	C=N^+^	C=N	π–π
S0, Pure PCL	73.9	15.2	10.9	0.0	0.0	0.0	0.0
S1, PPy-coated PCL	75.4	7.9	4.6	6.7	1.3	3.7	0.4
S2, Plasma treated PPy-coated PCL	48.6	2.7	1.7	38.3	4.2	4.2	0.3

**Table 3:** The percentage of different bonds at N 1 s spectrum for PPy-coated PCL without and with plasma treatment shows.

Sample	–NH–	–N=	C–N^+^	C=N^+^
S1, PPy-coated PCL fibers without plasma treatment	65.1	12.9	10.0	12.0
S2, PPy-coated PCL fibers with plasma treatment	79.2	1.8	0.0	19.0
